# Prolactin daily rhythm in suckling male rabbits

**DOI:** 10.1186/1740-3391-3-1

**Published:** 2005-01-13

**Authors:** Pilar Alvarez, Daniel Cardinali, Pilar Cano, Pilar Rebollar, Ana Esquifino

**Affiliations:** 1 Departamento de Biología Celular, Facultad de Medicina, Universidad Complutense de Madrid, 28040 Madrid, Spain; 2 Departamento de Fisiología, Facultad de Medicina, Universidad de Buenos Aires, 1121 Buenos Aires, Argentina; 3 Departamento de Bioquímica y Biología Molecular III, Facultad de Medicina, Universidad Complutense de Madrid, 28040 Madrid, Spain; 4 Departamento de Producción Animal, E.T.S.I. Agrónomos, Universidad Politécnica de Madrid, Spain

## Abstract

**Background:**

This study describes the 24-h changes in plasma prolactin levels, and dopamine (DA), serotonin (5HT), gamma-aminobutyric acid (GABA) and taurine concentration in median eminence and adenohypophysis of newborn male rabbits.

**Methods:**

Animals were kept under controlled light-dark cycles (LD 16:8, lights on at 08:00 h), housed in individual metal cages, and fed ad libitum with free access to tap water. On day 1 after parturition, litter size was standardized to 8–9 to assure similar lactation conditions during the experiment. Groups of 6–7 suckling male rabbits were killed by decapitation on day 11 of life at six different time points during a 24-h period.

**Results:**

Plasma prolactin levels changed significantly throughout the day, showing a peak at the beginning of the active phase (at 01:00 h) and a second maximum during the first part of the resting phase (at 13:00 h). Median eminence DA concentration also changed significantly during the day, peaking at the same time intervals as plasma prolactin. A single maximum (at 13:00 h) was found for adenohypophysial DA concentration. Individual adenohypophysial DA concentrations correlated significantly with their respective plasma prolactin levels. A maximum in median eminence 5HT concentration occurred at 21:00 h whereas adenohypophysial 5HT peaked at 13:00 h. Median eminence 5HT concentration and circulating prolactin correlated inversely. In the median eminence, GABA concentration attained maximal values at 21:00 h, whereas it reached a maximum at 13:00 h in the pituitary gland. Median eminence GABA concentration correlated inversely with circulating prolactin. In the median eminence, taurine values varied in a bimodal way showing two maxima, at the second half of the rest span and of the activity phase, respectively. In the adenohypophysis, minimal taurine levels coincided with the major plasma prolactin peak (at 01:00 h). Circulating prolactin and adenohypophysial taurine levels correlated inversely.

**Conclusion:**

The correlations among the changes in the neurotransmitters analyzed and circulating prolactin levels explain the circadian secretory pattern of the hormone in newborn male rabbits.

## Background

The mechanisms that regulate prolactin secretion are complex [1]. Two major regulatory inhibitory inputs for prolactin secretion are dopamine [2] and gamma-aminobutyric acid (GABA) [3-6]. In addition, many other neuromodulators have been implicated in the control of prolactin secretion, among them, vasoactive intestinal peptide, thyrotropin releasing hormone and serotonin (5HT) [1]. More recently, taurine has also been implicated in the regulation of prolactin secretion [1].

It is well known that basal secretion of prolactin varies throughout the day, describing a characteristic pattern with maximal values close to the light-dark transition [7,8]. Such a circadian pattern has been described not only in rodents (rat and mouse) but also in many other species [1]. In the rat, we previously demonstrated changes of the secretory pattern of prolactin along the year [7-11], as well as a function of aging [12,13].

The rat is very immature at birth, so that newborn and suckling rats are very sensitive to manipulations that can affect adulthood [14-18]. Circadian rhythms of developing mammals seem to be entrained by the rhythmicity of their mother [19,20], and several studies have indicated that maternal melatonin is necessary to entrain the circadian rhythms in the newborn [21,22].

The rabbit is probably the best-studied laboratory animal in the wild, due to its abundance, size and importance as an agricultural pest [23,24]. Wild and laboratory rabbits are essentially nocturnal and display a clear daily pattern of activity [25]. The rabbit possesses a number of behavioral specializations that make it uniquely suited for circadian studies. Female rabbits visit their altricial young only for a few minutes once every 24 h to nurse, and survival of the young depends on the tight circadian-controlled synchronization in behavior and physiology with the mother. This unusual pattern of maternal care and the demands it places on the litter provide an excellent opportunity to analyze circadian rhythms during early development [25].

In contrast to the large amount of information available on circadian rhythms in adult mammals, studies on circadian phenomena in neonates are few [26,27]. For example, in 21 day-old male rats the daily circadian pattern of prolactin secretion seen in adults is absent [18]. Considering that no information on circadian rhythmicity of prolactin secretion in neonatal male rabbits is available, we undertook the present study to analyze whether neonatal male rabbits show defined 24-h changes in plasma prolactin levels and whether neonatal male rabbits show circadian changes in DA, 5HT, GABA and taurine concentration in median eminence and the adenohypophysis, all of which are well known modulators of prolactin secretion.

## Methods

### Animals

This study was performed using 24 multiparous, lactating Californian × New Zealand White crossbreed doe rabbits. Animals were housed in research facilities of the Animal Production Department. They were maintained under controlled light-dark cycles (LD 16:8, light on at 08:00 h), housed in individual metal cages, fed at libitum using a commercial pellet diet (Lab Rabbit Chow, Purina Mills, Torrejón de Ardoz, Madrid, Spain) with free access to tap water. On day 1 after parturition, litter size was standardized to 8–9 by adding or removing kits to assure similar lactation conditions during the experiment. This study was performed according to the CEE Council Directive (86/609, 1986) for the care of experimental animals. Groups of 6–7 suckling male rabbits were killed by decapitation on day 11 of life at six different time points throughout a 24-hour cycle. The brains were quickly removed, and the median eminence and the anterior pituitary were taken out. Anterior pituitaries were weighed and homogenized in chilled (0–1°C) 2 M acetic acid. After centrifugation (at 15000 × g for 30 min, at 5°C), the samples were either analyzed for DA and 5HT or boiled for 10 min and further centrifuged at 14000 rpm for 20 min to measure GABA and taurine.

### Hormone assay

Plasma prolactin levels were measured by a specific homologous RIA method [28] using AFP-991086 antibody supplied by the National Institutes of Health (NIH, Bethesda, MD, USA) and Dr. A. F. Parlow (Harbour-UCLA Medical Center, CA, USA). The titer of antibody used was 1:62,500. The PRL standard used was RbPR_L_-RP-1. Hormone was labeled with ^125^I by the chloroamine-T method [29]. The volume of plasma for PRL determinations was 10 μl. *Staphylococcus aureus *(prepared by the Department of Plant Physiology, U.A.M., Madrid, Spain) was used to precipitate the bound fraction [28]. All samples were measured in the same assay run to avoid inter-assay variations. The sensitivity of the assay for PRL was 0.125 ng/ml and the intra-assay coefficient of variation was < 5%. The intra-assay coefficient of variation was calculated using a pool of plasma measured ten times in the same assay; mean (± S.E.M.) concentration was 106.9 ± 4.1 ng/ml.

### Catecholamine and indoleamine analysis

DA and 5HT concentration was measured by high pressure liquid chromatography (HPLC) using electrochemical detection (Coulochem, 5100A, ESA; USA), as described elsewhere [12]. A C-18 reverse phase column eluted with a mobile phase (pH 4. 0.1 M sodium acetate, 0.1 M citric acid, 0.7 mM sodium octylsulphate and 0.57 mM EDTA containing 10% methanol, v/v) was employed. Flow rate was 1 ml/min, at a pressure of 2200 psi. Fixed potentials against H_2_/H^+ ^reference electrode were: conditioning electrode: -0.4 V; preoxidation electrode: +0.10; working electrode: +0.35 V. Indoleamine and catecholamine concentration was calculated from the chromatographic peak heights by using external standards and was expressed as pg/μg protein. The linearity of the detector response for DA and 5HT was tested within the concentration ranges found in median eminence and adenohypophysial supernatants.

### Amino acid analysis

Amino acids were isolated and analyzed by HPLC with fluorescence detection after precolumn derivatization with O-phthalaldehyde (OPA) as described elsewhere [30]. An aliquot of the tissue supernatant containing homoserine as an internal standard was neutralized with 4 M NaOH and was then incubated at room temperature with OPA reagent (4 mM OPA, 10% methanol, 2.56 mM 2-mercaptoethanol, in 1.6 M potassium borate buffer, pH 9.5) for 1 min. The reaction was stopped by adding acetic acid (0.5 % v/v). Samples were immediately loaded through a Rheodyne (Model 7125) injector system (50 μl loop) to reach a C-18 reverse-phase column (4.6 mm ID × 150 mm, Nucleosil 5, 100A). Elution was achieved by means of a mobile phase consisting of 0.1 M sodium acetate buffer (pH 6.5) containing 35 % methanol, at a flow rate of 1 mL/min and a pressure of 140 Bars. The column was subsequently washed with the same buffer containing 70 % methanol and re-equilibrated with the elution buffer before re-use. The filter fluorometer was set at the following wavelengths: excitation: 340 nm, emission: 455 nm. The procedure allowed a distinct separation and resolution of the amino acids measured. Amino acid content was calculated from the chromatographic peak heights by using standard curves and the internal standard. The linearity of the detector response was tested within the concentration ranges found in median eminence and adenohypophysial extracts.

### Statistics

Statistical analysis of results was performed by a one-way analysis of variance (ANOVA) followed by post-hoc Tukey-Kramer's multiple comparisons tests. Curve estimation in regression analysis was made by using SPSS software, version 10.1 (SPSS Inc., Chicago, ILL). P values lower than 0.05 were considered evidence for statistical significance.

## Results

**Figure 1:**
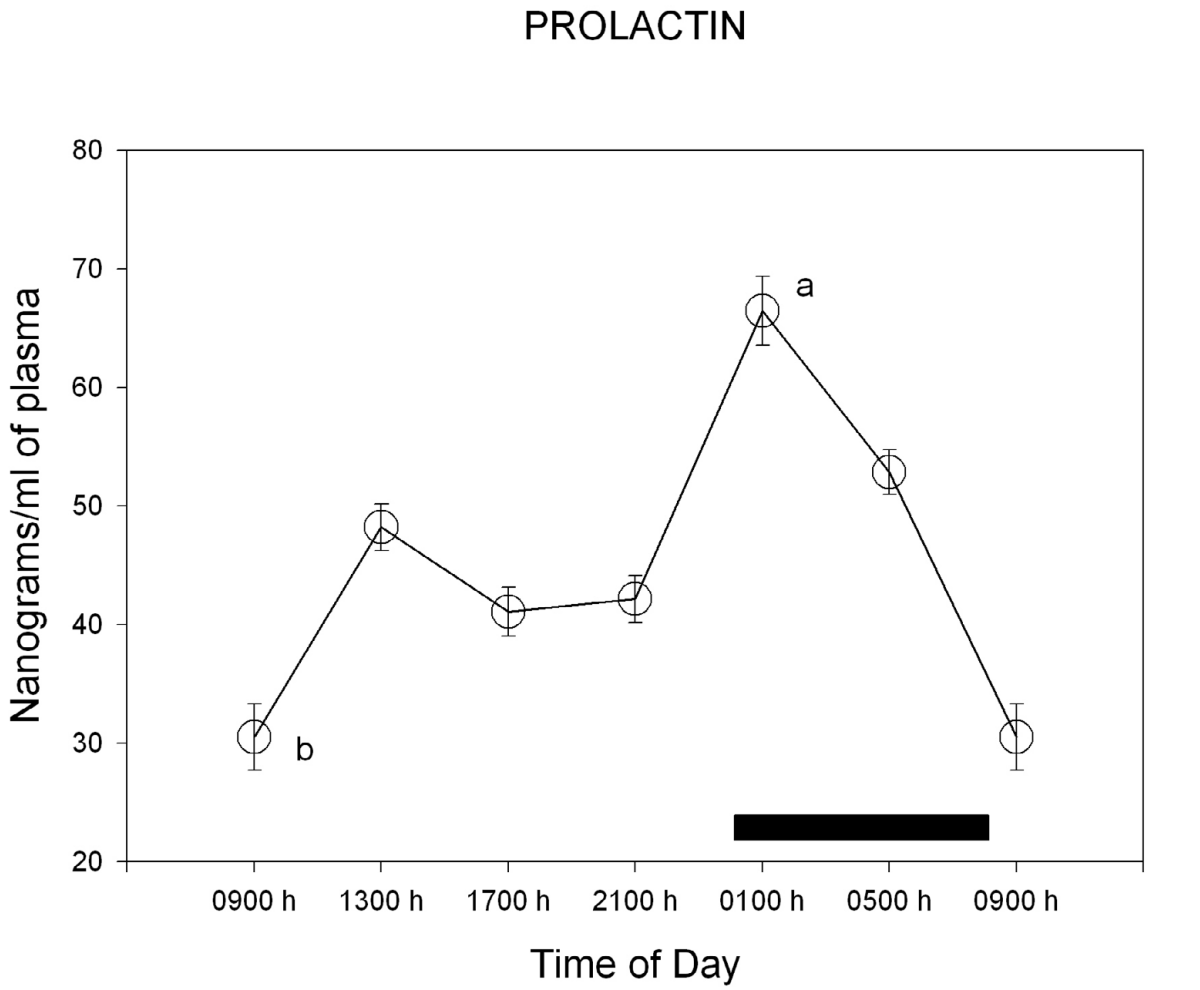
**24-h changes in plasma prolactin levels of 11 days old male rabbit pups. **Groups of 6–7 pups were killed by decapitation at 6 different time intervals throughout a 24 h cycle. Bar indicates scotophase duration. Results are the means ± SEM. ^a ^p < 0.01 vs. all time points. ^b ^p < 0.01 vs. 01:00 h, 05:00 h and 13:00 h, Tukey-Kramer's multiple comparisons test. For further statistical analysis, see text.

Figure 1 shows the levels of prolactin throughout the day in suckling male pups. Plasma prolactin levels changed significantly throughout the day (F = 21.1; p < 0.0001), showing two maxima, a major one at the beginning of the active phase (at 01:00 h) and a second one during the first part of the resting phase (at 13:00 h).

**Figure 2:**
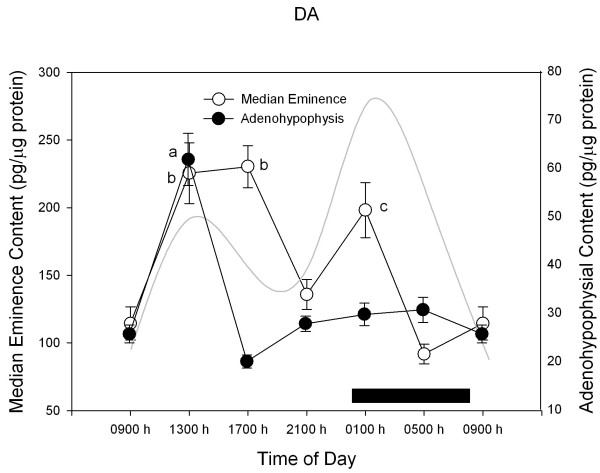
**24-h changes in median eminence and adenohypophysial DA concentration in 11 days old male rabbit pups. **Groups of 6–7 pups were killed by decapitation at 6 different time intervals throughout a 24 h cycle. Bar indicates scotophase duration. Results are the means ± SEM. Circulating prolactin levels are shown in shaded line. Letters indicate the existence of significant differences between time points within each tissue after a Tukey-Kramer's multiple comparisons test, as follows: ^a ^p < 0.01 vs. all time points. ^b ^p < 0.01 vs. 05:00 h, 09:00 h and 21:00 h. ^c ^p < 0.01 vs. 05:00 and 21:00 h. For further statistical analysis, see text.

**Figure 3:**
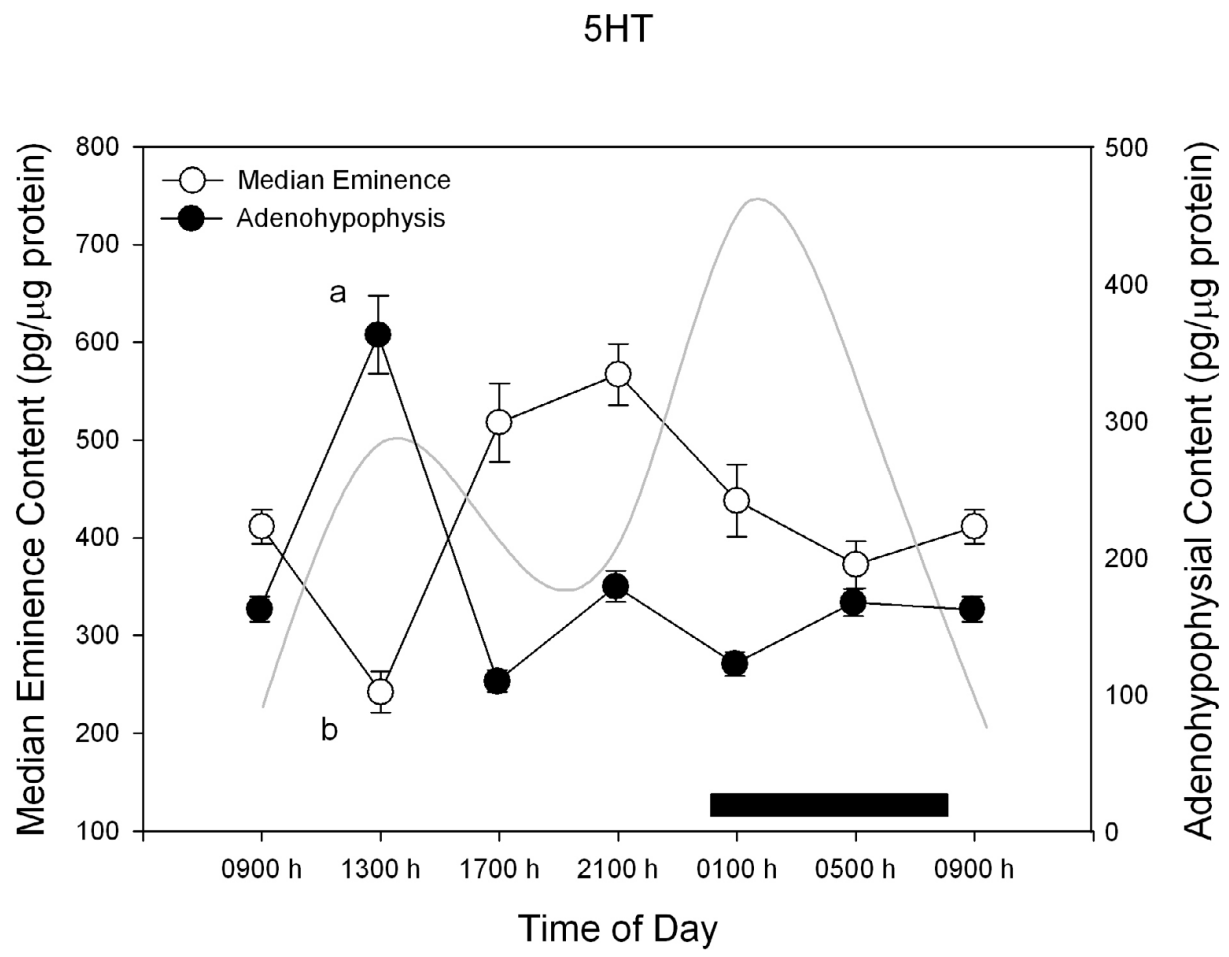
**24-h changes in median eminence and adenohypophysial 5HT concentration in 11 days old male rabbit pups. **Groups of 6–7 pups were killed by decapitation at 6 different time intervals throughout a 24 h cycle. Bar indicates scotophase duration. Results are the means ± SEM. Circulating prolactin levels are shown in shaded line. Letters indicate the existence of significant differences between time points within each tissue after a Tukey-Kramer's multiple comparisons test, as follows: ^a ^p < 0.01 vs. all time points. ^b ^p < 0.01 vs. 01:00 h, 09:00 h, 17:00 and 21:00 h. For further statistical analysis, see text.

**Figure 4:**
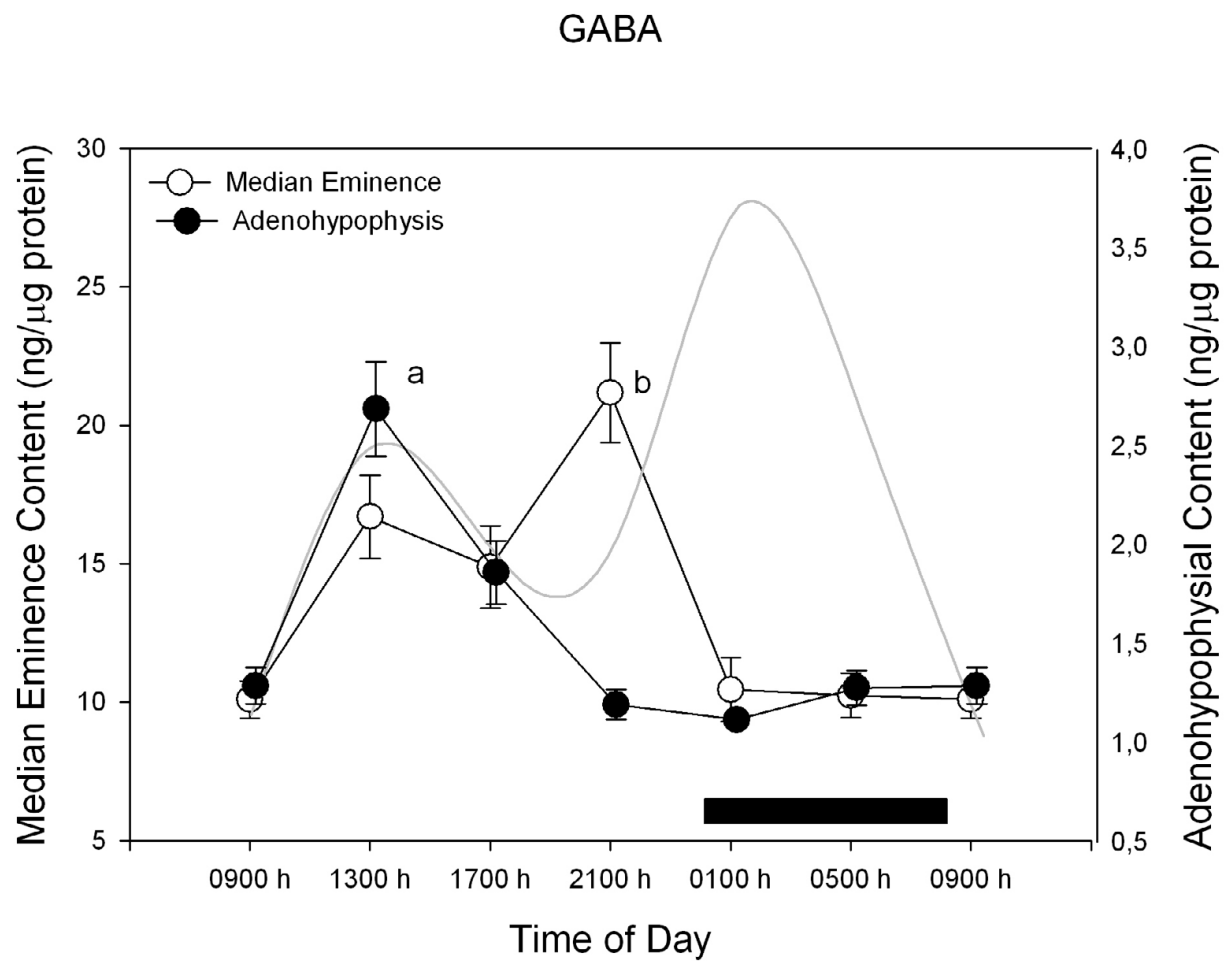
**24-h changes in median eminence and adenohypophysial GABA concentration in 11 days old male rabbit pups. **Groups of 6–7 pups were killed by decapitation at 6 different time intervals throughout a 24 h cycle. Bar indicates scotophase duration. Results are the means ± SEM. Circulating prolactin levels are shown in shaded line. Letters indicate the existence of significant differences between time points within each tissue after a Tukey-Kramer's multiple comparisons test, as follows: ^a ^p < 0.01 vs. all time points. ^b ^p < 0.01 vs. 01:00 h, 05:00 h and 09:00 h, p < 0.05 vs. 17:00 h. For further statistical analysis, see text.

**Figure 5:**
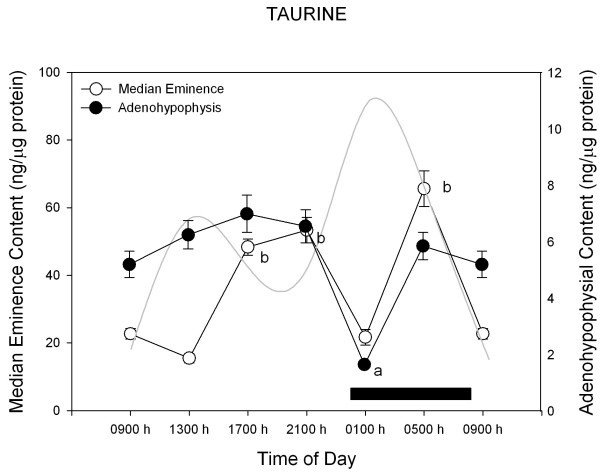
**24-h changes in median eminence and adenohypophysial taurine concentration in 11 days old male rabbit pups. **Groups of 6–7 pups were killed by decapitation at 6 different time intervals throughout a 24 h cycle. Bar indicates scotophase duration. Results are the means ± SEM. Circulating prolactin levels are shown in shaded line. Letters indicate the existence of significant differences between time points within each tissue after a Tukey-Kramer's multiple comparisons test, as follows: ^a ^p < 0.01 vs. all time points. ^b ^p < 0.01 vs. 01:00 h, 09:00 h and 13:00 h. For further statistical analysis, see text.

Figures 2,3,4,5 depict the changes in median eminence and adenohypophysial concentration of DA, 5-HT, GABA and taurine. Mean plasma prolactin concentration is plotted as a reference in every case.

Median eminence DA concentration changed in a bimodal way as a function of time of day, showing two maxima, coinciding with those of plasma prolactin at the active and resting phase of the diurnal cycle (F = 14.1; p < 0.0001, Figure 2). In the case of adenohypophysial DA concentration, a single maximum occurred during the first half of the rest phase (at 13:00 h) (F = 29.9; p < 0.0001). Only in the adenohypophysis, plasma prolactin and DA concentration correlated in a direct way. This correlation was best described by a log model with r^2 ^= 0.16, b_0 _= -123.7 and b_1_= 18.1 (F = 4.69, p= 0.04).

As shown in Figure 3, a maximum in median eminence 5HT concentration occurred at the second half of the rest span (F = 64.1; p < 0.0001) whereas a maximum in adenohypophysial 5HT levels was found at the first half of rest span. Circulating prolactin and median eminence 5HT concentration correlated inversely in a linear way (r^2^= 0.18, b_0 _= 677.6 and b_1 _= -4.9, F = 5.3, p < 0.03).

Figure 4 shows the changes in median eminence and adenohypophysial GABA concentration. In the median eminence, GABA concentration attained maximal values at the rest phase, with a peak at late evening (i.e. at 21:00 h, F = 11.1, p < 0.0001). In the anterior pituitary, GABA concentration reached a maximum at 13:00 h (F = 21.6, p < 0.0001). Circulating prolactin and median eminence GABA concentration correlated inversely in a linear way (r^2^= 0.21, b_0 _= 25.7 and b_1 _= -0.22, F = 6.6, p < 0.01).

Figure 5 depicts the 24-h changes in taurine concentration. In the median eminence, taurine values varied in a bimodal way showing a peak at the second half of the rest period, a nadir at the early activity span (coinciding with the prolactin peak) and a second maximum late in the activity phase (at 05:00 h, F = 32.9, p < 0.0001). Likewise, in the adenohypophysis, taurine levels exhibited minimal values at the time of the prolactin peak (i.e., at 13:00 h, F = 21.6, p < 0.0001). Circulating prolactin and adenohypophysial taurine levels correlated inversely in a linear way (r^2^= 0.42, b_0 _= 11.6 and b_1 _= -0.11, F = 17.4, p < 0.0001).

## Discussion

The present study, performed in neonatal male rabbit pups sacrificed at 6 different time intervals during a 24-h cycle, describes for the first time significant changes in plasma prolactin levels throughout the day. In concomitant measurements of median eminence and adenohypophysial concentration of DA, 5HT, GABA and taurine, a clear daily pattern was found in almost every case. Contrasting with neonatal rats that did not display any circadian pattern of plasma prolactin [18], a daily rhythm of plasma prolactin occurred in neonatal male rabbits, with a maximal value attained 1 h after lights-off (at 01:00 h) and a secondary peak found during the first part of the resting phase (at 13:00 h).

In adult rabbits, daily patterns of prolactin secretion depend on light/dark phases [25]. The present results indicate that, already on day 11 of life, male rabbit pups display daily changes in plasma prolactin levels, remarkably similar to those described in adult male rats (e.g., the maximum displayed 1 h after the dark onset) [7-10].

The activity of several nuclei of rabbit hypothalamus increases with age and with experience of anticipatory arousal [27]. However, no study has been published on the regulatory mechanism of prolactin in rabbits. Considering that DA is the major inhibitory input for prolactin secretion [1,32], the present study indicating that DA concentration in median eminence of rabbit pups is high during the rest phase of the day (when plasma prolactin levels are low), and decreases at day-night transition (coinciding with the increase in circulating prolactin), may support a cause-effect relationship. The afternoon decrease in median eminence DA concentration could be a prerequisite for prolactin release in neonatal male rabbits [2]. However, median eminence DA concentration of male rabbit pups also presents a peak during the activity phase (01:00 h) associated with the highest prolactin levels. Therefore, the data suggest that the inhibitory regulatory influence of DA on prolactin secretion is exerted mainly during the light phase of the photoperiod, whereas during the dark phase other hypothalamic neuromodulators could be operative, as it was previously described in rats [13]. These hypotheses must be tested rigorously (e.g., by using pharmacological blocking agents) before a definitive conclusion can be made.

Among other possible neuromodulators of prolactin secretion, the arcuate nucleus receives a dense serotonergic innervation consisting of a population of brainstem neurons arising mainly from the midbrain raphe nuclei [33] and from fibers originated in 5HT cell bodies located within the hypothalamus. There is a close proximity of 5HT fibers to dopaminergic cell bodies in the arcuate nucleus [34]. Therefore, an indirect effect of 5HT on prolactin release could be linked to the modulation of the inhibitory dopaminergic inputs to the pituitary. Our foregoing results agree with this hypothesis since 5HT concentration in median eminence changes diurnally in an opposite way to that of plasma prolactin levels, albeit without a significant correlation between them. Indeed, previous experiments in rats indicated that 5HT could probably modulate directly the secretion of prolactin [13].

Taurine has also been implicated in the regulation of prolactin release [5,13,35,36]. The foregoing results indicate that in median eminence and anterior pituitary of male rabbit pups taurine concentration varies inversely to plasma prolactin levels, displaying a mirror pattern. In the adenohypophysis a negative correlation between plasma prolactin and taurine levels was found, similarly to previous data obtained in rats [13]. Therefore, taurine may play a role in prolactin regulation in newborn rabbits.

A relatively dense innervation of GABA terminals exists in the external layer of the median eminence [37], and the ability of median eminence neurons to release GABA in portal blood has been demonstrated [38]. We previously demonstrated a possibly inhibitory control of GABA on prolactin secretion during the activity phase in male rats [3-6]. Results obtained in the present study in suckling male rabbits support such an inhibitory effect of GABA on plasma prolactin levels exerted mainly during the dark phase of daily photoperiod. The data indicate that GABA concentration in median eminence decreased during the day-night transition, while plasma prolactin levels were increasing. Actually, in median eminence a negative correlation between GABA concentration and plasma prolactin was found, thus suggesting an inhibitory effect of GABA on prolactin secretion.

GABA acting on specific receptors in the anterior pituitary has been reported to suppress prolactin secretion [39,40], although whether this effect was physiological has been questioned [40]. Data from literature suggest that the role of GABA on prolactin release is quite complex [41]. In some conditions, such as aging [13] or hyperprolactinemia [6], the inhibitory role of GABA becomes more pronounced whereas the inhibitory control exerted by DA diminishes. Our results in male rabbit pups indicated that, although no correlation between plasma prolactin and pituitary GABA concentration was found, the pattern may confirm the main role of this amino acid in the control of prolactin secretion during the dark phase of the photoperiod that was developed later. Again, all these hypotheses must be tested. e.g. pharmacologically, before a definitive conclusion on this matter can be drawn.

## Conclusions

In suckling male rabbits plasma prolactin and median eminence and anterior pituitary concentration of several neuromodulators change on a daily basis. The existence of significant correlations among several of the neurotransmitters analyzed and plasma prolactin levels may explain the circadian secretory pattern of prolactin at this age in suckling rabbits. Collectively, the present results differ from the reported absence of circadian rhythmicity of prolactin and median eminence and adenohypophysial neuromodulators in rats at a comparable age.

## Competing Interests

The author(s) declare that they have no competing interests.

## Authors' Contributions

MPA and PC carried out the experiment and the immunoassays and the analysis of catecholamines, indoleamines and amino acids. DPC and AIE designed the experiments. Also, DPC performed the statistical analysis. PR took care of the experimental animals. AIE supervised its technical implementation and drafted the manuscript. All authors read and approved the final manuscript.
